# Reliable Determination of ATP and Its Metabolites by LC-MS Using Blood Collection Tubes with and without Ectonucleotidase Inhibitors

**DOI:** 10.1021/acsptsci.5c00446

**Published:** 2025-11-07

**Authors:** Riekje Winzer, Johanna Hiefner, Romy Hackbusch, Moritz A. Link, Götz Thomalla, Eva Tolosa, Anna Worthmann

**Affiliations:** † Department of Immunology, 37734 University Medical Center Hamburg-Eppendorf, 20246 Hamburg, Germany; ‡ Department for Biochemistry and Molecular Biology, University Medical Center Hamburg-Eppendorf, 20246 Hamburg, Germany; § Department of Neurology, 37734 University Medical Center Hamburg-Eppendorf, 20246 Hamburg, Germany

**Keywords:** Adenine nucleotides, Adenosine metabolism, ATP metabolism, LC-MS/MS, Blood sampling

## Abstract

Adenosine triphosphate (ATP) and its breakdown products, including adenosine, play key roles in regulating immune responses. Altered ATP and adenosine levels in blood may reflect the presence or development of various pathologies; however, their rapid metabolism and clearance makes accurate measurement of their concentrations difficult. Not surprisingly, studies simultaneously monitoring ATP and its breakdown products are sparse and show conflicting results, and the workflows used are difficult to implement in clinical routine. Here, we present the simultaneous measurement of ATP and its metabolites in blood samples from healthy donors by combining a liquid chromatography–mass spectrometry-based quantification method with various procedures of blood sampling. We find that ATP and adenosine are best preserved in an ethylenediaminetetraacetic acid (EDTA) blood collection tube containing ectonucleotidase and nucleoside transporter inhibitors. In contrast, inosine and its downstream metabolites are detected in a serum collection tube without inhibitors. Therefore, we propose the use of these two sampling tubes to obtain a faithful determination of ATP and its degradation products. Overall, our approach provides a valuable and reliable tool to monitor changes in the concentration of ATP metabolites that can be easily implemented for biobanking purposes in the context of clinical trials.

## Graphical abstract

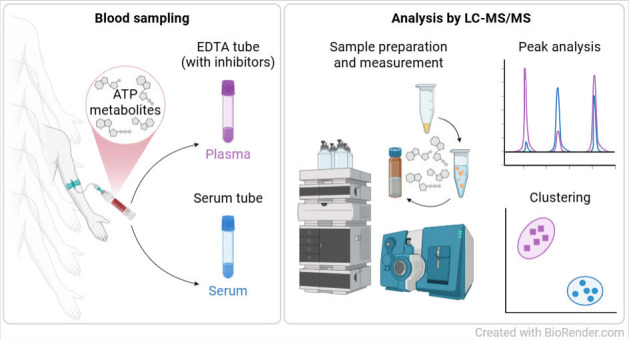

Cells harbor millimolar concentrations of intracellular adenosine triphosphate (ATP) within the cytosol, which can be rapidly released into the extracellular milieu in response to stress or inflammatory stimuli. Extracellular ATP acts as a danger signal, engaging purinergic P2 receptors, specifically ionotropic P2X and metabotropic P2Y subtypes, to orchestrate diverse immunomodulatory effects, predominantly skewed toward proinflammatory signaling pathways. The concentration of ATP in blood changes rapidly because of a continuous release from activated or damaged cells and efficient hydrolysis by membrane-bound or soluble ectonucleotidases. Ectonucleoside triphosphate diphosphohydrolase (ENTPD) and ectonucleotide pyrophosphatase/phosphodiesterase (ENPP) family members are responsible for the degradation of ATP to adenosine monophosphate (AMP), and AMP can then be further metabolized to adenosine by CD73.^1,2^ In contrast to ATP, adenosine has anti-inflammatory effects on immune cells,^3^ highlighting the importance of a strictly regulated balance of ATP and adenosine. Adenosine has a very short half-life of less than ten seconds in blood because it is rapidly cleared.^4^ This happens predominantly through its rephosphorylation to AMP by adenosine kinase (ADK), by degradation to inosine mediated by adenosine deaminases (ADA), or by uptake into cells through equilibrative nucleoside transporters (ENTs).^5^ Inosine may be further degraded to hypoxanthine, xanthine, and uric acid.

The concentration of adenine nucleotides in blood changes under certain physiologic and pathologic conditions, and the measurement of the concentrations could give an estimation of the immune regulatory status of an organism. Extracellular ATP concentrations increase systemically after the injection of lipopolysaccharide (LPS) in mice^6^ and are locally high in the tumor microenvironment.^7^ In a mouse model of stroke, ATP is rapidly released after stroke induction, with increased ATP concentrations still detectable after 24 h.^8^ In patients with stroke or transient ischemic attack (TIA), adenosine levels in peripheral blood are increased in the acute phase (2–3 days after stroke or TIA) compared to later time points,^9^ probably due to the degradation of the released ATP. This suggests a shift toward an anti-inflammatory environment, concordant with reported poststroke immune suppression.^10^ Brain injury and epilepsy also yield higher purine concentrations in blood.^11,12^ Further, adenosine concentrations, as well as ATP and adenosine diphosphate (ADP) concentrations, are higher after physical exercise^13–15^ and caffeine intake.^16^

Due to their rapid turnover in blood, the measurement of adenine nucleotides has proven to be difficult. One method to assess the dynamic changes in adenine nucleotides in plasma or serum is the measurement of the enzymatic activities of ectonucleotidases involved in the metabolism of ATP to inosine and further enzymes responsible for the degradation of inosine to uric acid. Another possibility is the direct measurement of adenine nucleotide concentrations, for example, by high performance liquid chromatography (HPLC) or mass spectrometry (MS). The quantification of adenine nucleotides can be optimized by preventing their metabolism, for example, by rapid chilling of the samples^17^ or by blocking metabolism and uptake using inhibitors.^18,19^ Fluorescent labeling of adenine nucleotides further increases the sensitivity of the measurement.^17^ The reported concentrations of adenine nucleotides in serum or plasma vary considerably across different studies. For instance, the concentration of ATP ranges between 200 nM and 13.2 μM in ethylenediaminetetraacetic acid (EDTA) plasma and is lower in heparin plasma (reported values of <40 nM to 3.8 μM) and serum (0.8 μM).^13,17,20^ The concentration of adenosine is around 80 nM in heparin plasma and 7.7 μM in serum.^13,17^

Assessment of changes in the concentration of adenine nucleotides in circulation could help one to understand the course of disease and evaluate the response to therapy. For example, patients treated with the P2Y12 inhibitor Ticagrelor show higher adenosine concentrations in blood because the adenosine uptake by ENTs in red blood cells is inhibited.^18,21^ Here, we established a pipeline for detecting adenine nucleotides and their degradation products in blood samples using a highly sensitive LC-MS-based approach. We find dramatic differences in the concentration of ATP metabolites, depending on the blood collection tube. While inosine and its downstream metabolites can be detected in serum, determination of ATP and adenosine requires the presence of ectonucleotidase inhibitors in the blood collection tube. Our data suggest that two types of collection tubes, one without any inhibitor and one containing specific ectonucleotidase inhibitors in addition to EDTA, are necessary for the determination of the whole range of ATP metabolites. Importantly, our protocol is suitable for application in biobanking of clinical cohorts with low laboratory effort and few sources of error. The results of our study will help in the design and performance of measurements of adenine nucleotides in blood samples from healthy donors and patients.

## Methods

### Origin of Samples

Peripheral blood (PB) was obtained from healthy volunteers (seven males and eight females in the age range of 23–73 years) and individuals with high cardiovascular risk. The ethics protocols were approved by the Ethics Committee of the Hamburg Chamber of Physicians (refs PV5139 and PV6051). Sex and age distributions of the healthy donor cohort and of the cohort of patients with high cardiovascular risk are shown in Supporting Figure 1A,B.

### Sample Preparation

PB of each donor was drawn into blood collection tubes containing EDTA, Lithium-Heparin Gel, or Serum Gel (all S-Monovette, Sarstedt). In addition, blood was collected in a 2.7 mL EDTA tube with 1.3 mL of an inhibitor mix containing 15 mM EDTA (Applichem), 100 μM erythro-9-(2-hydroxy-3-nonly)­adenine (EHNA) (Cayman Chemicals), 100 μM 5-iodotubercidin (Merck), 40 μM dipyridamole (Merck), 150 μM ARL 67156 (Tocris Bioscience), 220 μM adenosine 5′-(α,β-methlyene)­disphosphate sodium salt (AOPCP, Tocris Bioscience), and 100 μM *S*-(4-nitrobenzyl)-6-thioinosin (NBMPR) (Merck) in PBS (Gibco).^18^ The inhibitor mix was prepared in ready-to-use aliquots and frozen at −20 °C. It was added to the EDTA tubes prior to blood collection under sterile conditions, leading to a 1.48-fold dilution of the blood.

### Isolation of Plasma and Serum

Plasma was isolated from EDTA, EDTA+inhibitors, and Lithium-Heparin tubes by centrifugation at 500*g* for 10 min (room temperature, RT). The plasma was collected and centrifuged again at 13,000*g* for another 10 min (RT). Serum was isolated from the Serum Gel tube by centrifugation at 2,500*g* for 10 min (RT). Serum and plasma were aliquoted and stored at −70 °C.

### Reference Standards

HPLC-grade ATP, ADP, AMP, adenosine, inosine, hypoxanthine, xanthine, and uric acid were all obtained from Sigma-Aldrich. Stock solutions of these metabolites (1 mg/mL in water) were prepared on ice, stored at −20 °C, and used for a maximum of three months. A 80 μM multistandard solution was prepared for the calibration curve and diluted into 13 calibration levels ranging from 0.002 to 20 μM in acetonitrile shortly before the measurement.

### Sample Extraction

For extraction of metabolites of the ATP to uric acid axis, 10 μL of plasma or serum were transferred to ice-cooled 1.5 mL reaction tubes. Samples were spiked with 10 μL of ice-cooled 200 μM ^13^C_6_-nicotinamide (Sigma-Aldrich) as an internal standard (IS), and 180 μL of cold acetonitrile/water ((80/20); (v/v)) was then added to the samples. For homogenization, plasma and serum samples were vortexed at the highest level for 1 min, followed by incubation on ice for 10 min. The samples were centrifuged at 4 °C and 16,000*g* for 10 min. The supernatant was carefully transferred to an amber glass vial (Phenomenex), which was tightly closed with a crimped cap (Labsolute). The extracts were measured immediately after extraction and then stored at −80 °C.

### High Performance Liquid Chromatography-Tandem Mass Spectrometry (HPLC-MS/MS)

All HPLC-MS/MS analyses were performed on a triple quadrupole mass spectrometer (QTRAP 5500; SCIEX) coupled to an ultrahigh pressure liquid chromatography system (Nexera X2; Shimadzu). Analyte separation was performed using the HPLC column Luna 3 mm NH2 100 Å 150 mm × 2 mm (Phenomenex) in hydrophilic interaction liquid chromatography (HILIC) mode as a stationary phase. The instrument was operated with Analyst Software 1.7 (Sciex). The mass spectrometer was equipped with an ESI source and run in multiple reaction monitoring (MRM) mode. All measurements were carried out in negative ionization mode with the source temperature set to 500 °C with an ion spray voltage of −4500 V. Collision gas was set to medium; ion source gases 1 and 2 were set to 40 and 20, respectively, and curtain gas was set to 20. Optimized MS parameters including MRM transitions for each analyte are given in Table 1. The HPLC system was equipped with a degasser unit, a binary pump, and a cooled autosampler. The autosampler temperature was set to 4 °C for all of the measurements. Eluent A contained 20 mM ammonium acetate in MS-grade water (pH 9.8), and solvent B contained 100% acetonitrile. The flow rate was set to 0.25 mL/min with a linear gradient of decreasing solvent B (0–17 min, 80–0%), followed by 8 min at 0% solvent B and 5 min re-equilibration at 80% solvent B.

**Table 1:** Mass Spectrometry Parameters and Retention Time of Metabolites of the ATP to the Uric Acid Axis^a^

Q1 [Da]	Q3 [Da]	ID	DP [V]	EP [V]	CE [V]	CXP [V]	RT [min]
505.953	79.000	ATP	–150	–10	–96	–9	19.16
425.980	79.000	ADP	–100	–10	–80	–11	16.66
346.064	78.900	AMP	–110	–10	–70	–9	13.39
266.105	134.100	Adenosine	–135	–10	–28	–15	3.77
266.976	134.800	Inosine	–105	–10	–28	–13	6.51
134.909	91.900	Hypoxanthine	–95	–10	–22	–11	6.23
151.000	107.900	Xanthine	–90	–10	–22.5	–10.5	8.77
202.948	116.000	Uric acid	–90	–10	–21	–7.5	5.43
127.055	43.100	^13^C NAM	–95	–10	–36	–21	2.48

a DP, Declustering potential; EP, Entrance potential; CE, Collision energy; CXP, Collision exit potential; RT, Retention time; NAM, Nicotinamide.

### Data Processing and Analysis

Raw MS data was analyzed using SCIEX OS software (Sciex) to generate quantitation reports containing analyte peak areas. Quantification and calculation of absolute concentrations were done manually in Microsoft Excel using linear regression of the standard calibration curves to quantitate accurate concentrations that reflect the analytical run performance. Prior to quantitation, all analyte areas were normalized against the area of the IS ^13^C_6_-nicotinamide. Calibration levels for quantitation were adapted to low or high concentration ranges when needed according to a previous method validation. Intrameasurement variability was accounted for by running calibration standard levels before and after the samples. Accurate analyte concentrations were calculated by using a minimum of six points per calibration curve.

### Data Representation and Statistics

Data representation and statistical analysis were performed using Prism 10 (GraphPad) and R (version 4.4.2). Dimensionality reduction was performed using the UMAP (Uniform Manifold Approximation and Projection) algorithm (R package “umap”) to visualize the metabolite data of all donors in a two-dimensional space. The Mann–Whitney test was used for comparisons between two groups with unpaired, nonparametric data. For comparisons between two groups with paired, nonparametric data, the Wilcoxon test was used. For comparisons among multiple groups with paired, nonparametric data, the Friedman test was used in combination with Dunn’s multiple comparisons post hoc test. Detailed information regarding the statistical tests is provided in the figure legends. Statistical significance is indicated as *p ≤ 0.05, **p ≤ 0.01, ***p ≤ 0.001, and ****p ≤ 0.0001. Nonsignificant differences are not annotated.

## Results

### Establishment of a Sensitive LC-MS-Based Method to Reliably Quantify ATP and Its Metabolites

We set out to develop a sensitive and reliable LC-MS-based method for the simultaneous quantification of ATP and its breakdown products in human blood. To provide a holistic view on these metabolites, we expanded our recently published LC-MS-based method for the accurate quantification of adenine nucleotides^22^ and included the determination of xanthine and uric acid concentrations. We obtained a clear chromatographic separation of all analytes (Figure 1A) with inosine showing the most intense peak (Figure 1A, upper panel) and xanthine giving the lowest signal intensity (Figure 1A, lower panel, zoom in). Even though signal intensity varied among the metabolites, validation of the additionally implemented metabolites xanthine and uric acid confirmed the accurate quantification over a broad concentration range from nano- to micromolar values. Validation parameters for xanthine and uric acid are shown in Supporting Table 1.

**Figure 1:**
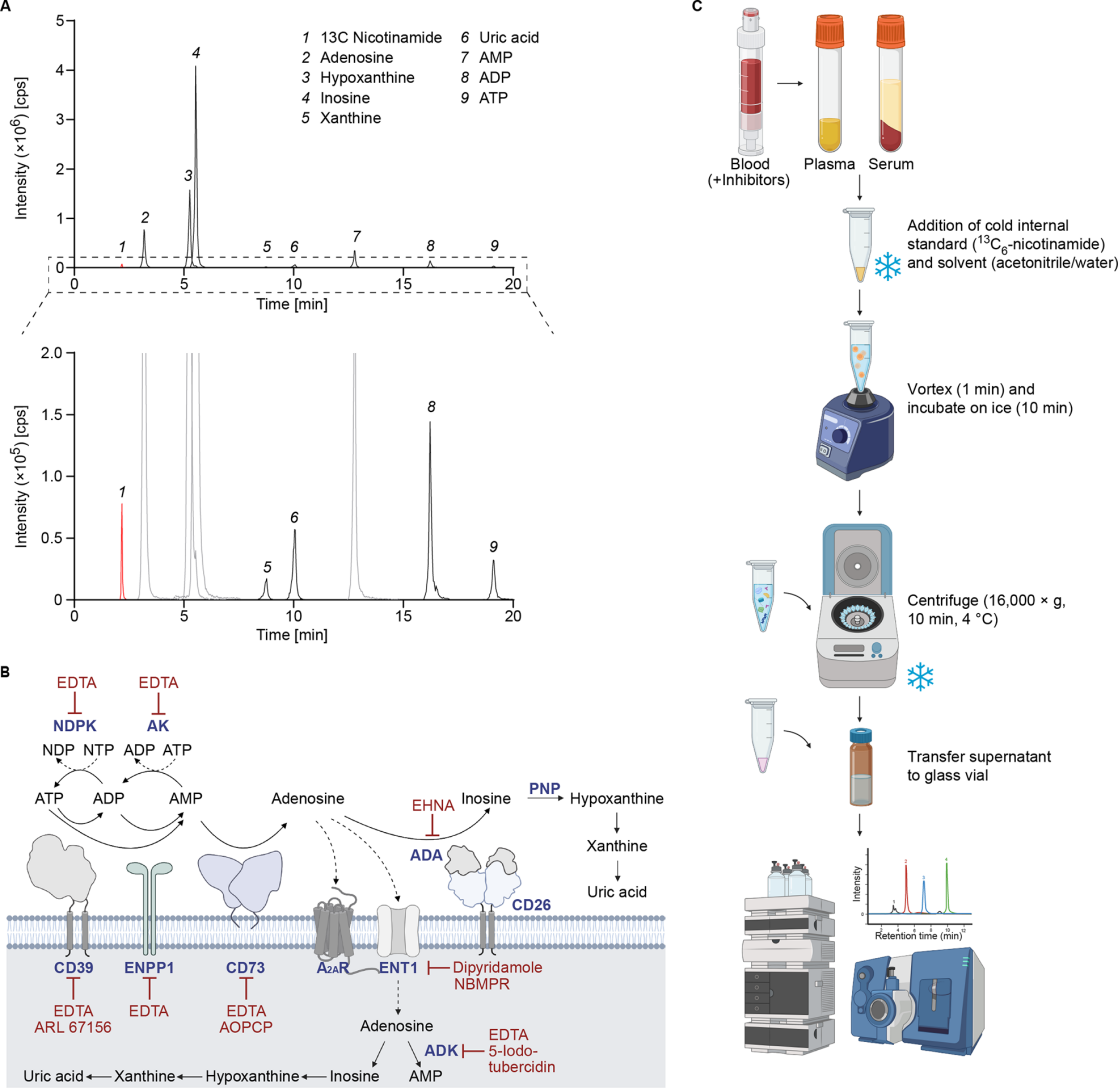
Pipeline for the quantification of ATP and its metabolites by LC-MS. (A) Chromatogram corresponding to the 2 μM multistandard solution containing: 1, ^13^C Nicotinamide (internal standard, labeled in red); 2, Adenosine; 3, Hypoxanthine; 4, Inosine; 5, Xanthine; 6, Uric acid; 7, AMP; 8, ADP; 9, ATP (upper panel); its magnification (lower panel). (B) Scheme of the enzymes and transporters involved in the metabolism of ATP to uric acid. Red bars indicate the enzyme and nucleoside transporter inhibitors present in the “inhibitor tube” (inhibitor cocktail adapted from Löfgren et al.^18^). (C) Blood collection tubes and sample processing protocol. ADA, Adenosine deaminase; ADK, Adenosine kinase; ADP, Adenosine diphosphate; AK, Adenylate kinase; AMP, Adenosine monophosphate; ATP, Adenosine triphosphate; EDTA, Ethylenediaminetetraacetic acid; EHNA, erythro-9-(2-hydroxy-3-nonly)­adenine; ENT, Equilibrative nucleoside transporter 1; NDP, Nucleoside diphosphate; NDPK, Nucleoside diphosphate kinase; NTP, Nucleoside triphosphate; PNP, Purine nucleoside phosphorylase. Schematic representations were created with BioRender.com.

The turnover of extracellular adenine nucleotides is extremely dynamic.^5^ In the bloodstream, extracellular ATP is degraded to ADP and AMP by CD39 or to AMP and PPi by ENPP1. After conversion of AMP to adenosine by CD73, adenosine may be cleared from the bloodstream by nucleoside transporter ENT1-dependent uptake into the cell or by ADA-mediated catabolism. The resulting inosine is further metabolized to hypoxanthine (mediated by purine nucleoside phosphorylase (PNP)), xanthine, and the stable end product uric acid. Adenosine may be used for ATP regeneration by backward ecto-phosphotransfer reactions mediated by adenosine kinase (ADK), adenylate kinase (AK), and nucleoside diphosphate kinase (NDPK)^23,24^ (Figure 1B).

To analyze adenine nucleotides or other metabolites in the bloodstream, their concentrations can be quantified in plasma or serum. These biological fluids, however, differ significantly because of the absence or presence of anticoagulants and the preparation process, e.g., centrifugation speed. As there is no agreement on the physiologic blood adenine nucleotide levels and there are different methods to preserve blood levels of ATP and/or adenosine, we decided to systematically compare adenine nucleotide concentrations in blood samples in a cohort of healthy volunteers. For each individual, we prepared serum samples, plasma samples using heparin- or EDTA-plasma tubes, and EDTA-plasma tubes that were preincubated with an inhibitor cocktail (EDTA+I) designed to preserve adenine nucleotide concentrations (Figure 1B), described by Löfgren et al.^18^ This inhibitor cocktail contained EDTA, which complexes divalent cations, thereby inhibiting CD39, CD73, and ENPP1, as well as other enzymes (e.g., NDPK), which use divalent cations as cofactors. CD39 and CD73 activities were further blocked by ARL67156 and AOPCP, respectively. ENT1-mediated nucleoside transport was inhibited by dipyridamole and NBMPR, and degradation of adenosine was prevented using EHNA to block ADA and 5-iodotubercidin to prevent ADK activity. A summary of all used inhibitors and their effects is depicted in Figure 1B.

The extraction of nucleotides from serum and three different plasma types was performed on ice by adding internal standard solution and prechilled acetonitrile/water (80/20) to samples. Following homogenization and centrifugation at 4 °C, we transferred the supernatant to glass vials and subsequently subjected them to LC-MS analysis (Figure 1C).

### The Concentration of Metabolites Detected in Blood Depends on the Blood Collection Tubes Used

To get a first impression of the similarities and differences in the concentration of ATP metabolites in the different individuals according to the blood collection tube, we used dimensionality reduction (Uniform Manifold Approximation and Projection, UMAP) to project the concentration of ATP metabolites for each of the 15 individuals and each of the four blood collection tubes, resulting in a total of 60 data points. The UMAP projection revealed a clear separation between serum and the three plasma sample types, with the serum samples (depicted in blue) forming a distinct cluster (Figure 2A, upper panel). The variation was lower among the three plasma samples, with the heparin plasma samples (purple) tending to cluster separately from the EDTA plasma (Figure 2A, middle panel). Finally, the EDTA plasma (green) and EDTA+I plasma (yellow) still formed two distinguishable clusters (Figure 2A, lower panel).

**Figure 2:**
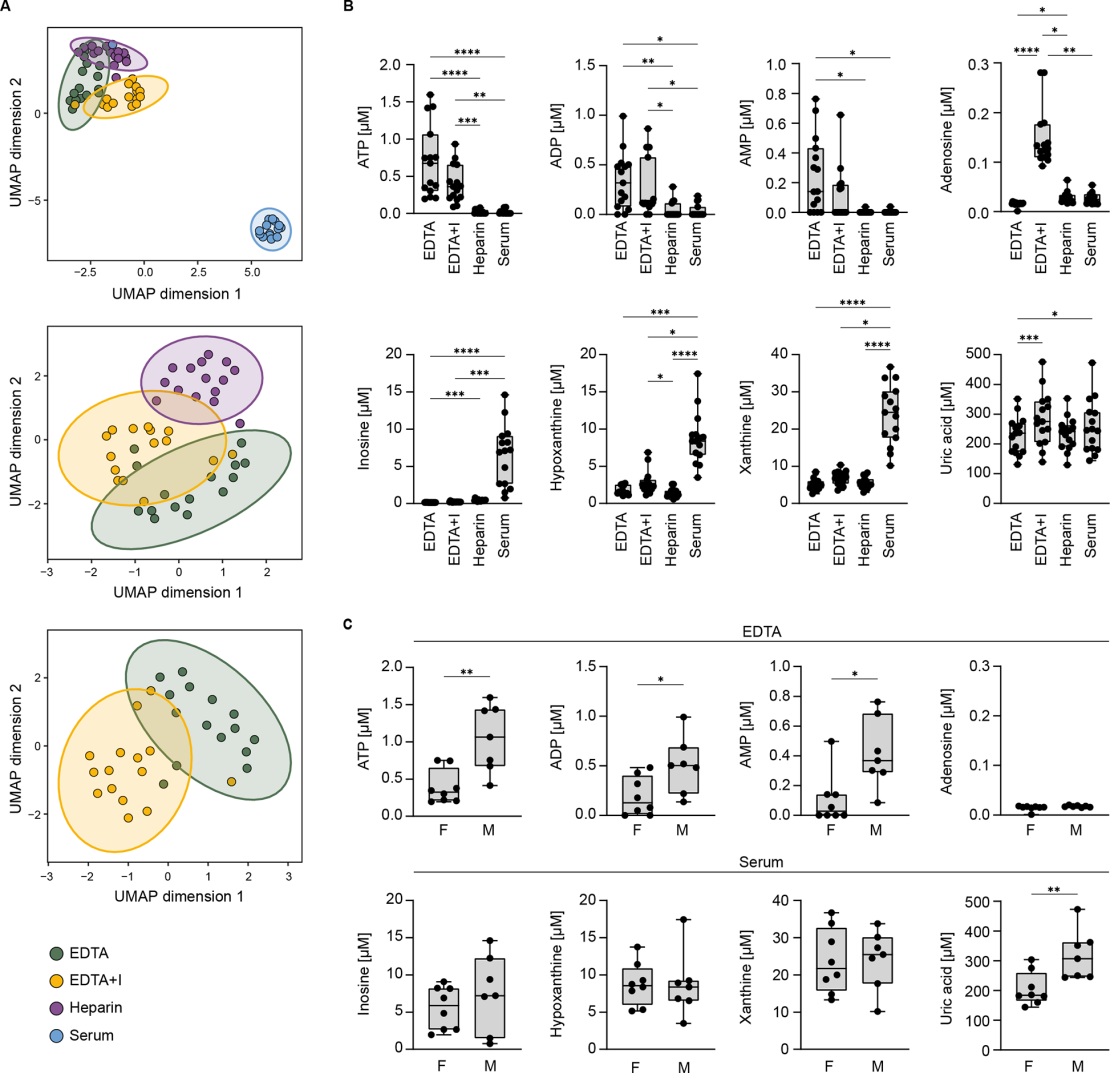
Blood collection tube additives affect the concentrations of ATP metabolites in human samples. Metabolites of the ATP to uric acid axis were measured in different blood collection tubes by LC-MS (*n* = 15). (A) Dimensionality reduction (UMAP) to visualize global differences between the blood collection tubes (EDTA = green; EDTA+I = yellow, Heparin = purple, Serum = blue). Each dot represents the ATP metabolite concentration per individual and sample collection tube (in total 60 dots for 4 sample tubes and 15 individuals). (B) Concentrations of each analyte, separated by blood collection tubes. (C) Differences between the concentration of selected metabolites between females and males. The Friedman test with Dunn’s multiple comparison test was used to compare the concentration of metabolites between several groups in (B) and the Mann–Whitney test was used to compare the differences between two groups in (C) (*p ≤ 0.05, **p ≤ 0.01, ***p ≤ 0.001, ****p ≤ 0.0001). F, female; M, male; UMAP, uniform manifold approximation and projection.

When comparing the levels of individual metabolites, we detected ATP, AMP, and ADP mainly in samples from the EDTA or EDTA+I tube but not in samples derived from heparin and serum tubes. In samples from EDTA+I tubes, we detected median ATP concentrations of 0.36 μM and median ADP levels of 0.12 μM and AMP was below the detection limit (Figure 2B). Interestingly, even though not significant, ATP levels were nearly doubled in plasma derived from EDTA-only tubes compared to EDTA+I tubes (0.68 versus 0.36 μM, respectively). As expected, adenosine levels were highest in the plasma from the EDTA+I tube, containing the ADA inhibitor EHNA and inhibitors of ENT1, compared to all other tubes (Figure 2B). The median adenosine concentration was 0.13 μM in EDTA+I tubes, around 5-fold higher than in all other tubes (<0.03 μM). Of note, we detected significantly more adenosine in heparin plasma than in EDTA plasma (Figure 2B). Further, adenosine degradation products, i.e., inosine, hypoxanthine, and xanthine, were best detected in the serum tube. Compared to their concentration in all plasma samples, serum contained the highest concentrations of inosine (7.1 μM), hypoxanthine (8.4 μM), and xanthine (24.5 μM). Uric acid was detected in all collection tubes used at a concentration of approximately 250 μM. The data from Figure 2B are summarized in Supporting Table 2.

We also analyzed whether there are sex-specific differences in the concentration of these metabolites. We found that men have higher concentrations of ATP, ADP, and AMP in EDTA plasma (Figure 2C); however, these differences could not be detected in serum samples or in plasma derived from heparin or EDTA+I tubes. As previously reported, the concentration of uric acid was higher in men than in women in all collection tubes analyzed (Figure 2C and Supporting Figure 2A,B).^25^

In summary, we find that the concentration of adenine nucleotides in blood is dependent on the type of collection tube used for sampling. ATP, ADP, and AMP can only be detected in collection tubes containing EDTA, while determination of adenosine required the addition of specific inhibitors of ADA and nucleoside transporters. In contrast, the adenosine degradation products are best detected in serum collection tubes. The concentration of uric acid is reliably determined in all tubes and is higher in males than in females.

### The Pattern of ATP Metabolite Concentrations Across Different Blood Collection Tubes Is Preserved in Samples from a Clinical Study

Next, we aimed to broaden the focus of our study from basic research to clinical applications. To this end, we performed a pilot study to assess the applicability of the blood sampling in EDTA+I tubes in a daily clinical routine. Blood from patients with high cardiovascular risk was collected into serum tubes as well as EDTA tubes containing the inhibitor mix and then subjected to our LC-MS method. Even though we did not standardize the time and temperature conditions from the moment of blood withdrawal until the centrifugation of the samples, we were still able to detect ATP, ADP, AMP, and adenosine in the samples derived from tubes containing EDTA and the inhibitor cocktail (Figure 3). In line with our previous findings, in serum samples of the same patients, we did not detect ATP or adenosine at all and detected only low concentrations of ADP and AMP. In contrast, the degradation products downstream of adenosine such as inosine, hypoxanthine, and xanthine were detected in serum (Figure 3). Therefore, our sampling can be easily implemented in clinical settings.

**Figure 3:**
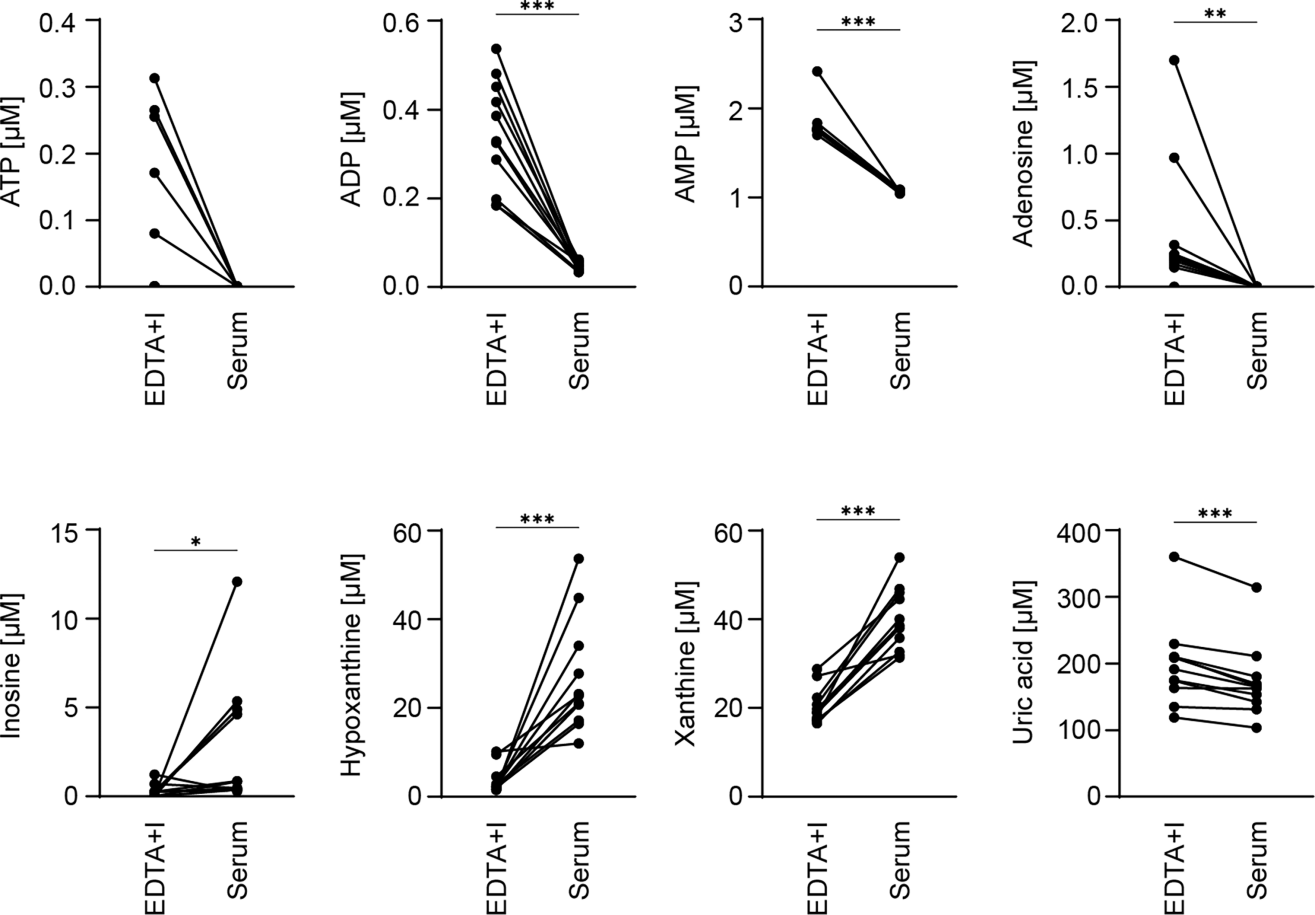
Clinical application of EDTA+Inhibitor and serum blood collection tubes in a cohort of patients with a high risk of cardiovascular disease. Metabolites of the ATP to uric acid axis were measured in EDTA+Inhibitor (EDTA+I) and serum tubes by LC-MS (*n* = 11). Analyte concentrations of each patient, separated by blood collection tubes. The Wilcoxon test was used to compare the differences in metabolite concentrations between the two groups (*p ≤ 0.05, **p ≤ 0.01, ***p ≤ 0.001, ****p ≤ 0.0001).

## Discussion

The concentrations of ATP and its metabolites in blood could be indicative of the presence or development of pathologies. The rapid turnover of the metabolites makes their reliable measurement difficult, resulting in inconsistent data in the literature and unknown baseline concentrations. We describe an easy and rapid approach to quantify metabolites of the ATP to uric acid axis in human blood and found that the concentrations of ATP metabolites are highly dependent on the blood collection tube additives. The combination of an EDTA tube preincubated with a mix of inhibitors and a serum tube allows for the determination of ATP and its degradation products down to uric acid.

In the bloodstream, the balance between extracellular ATP and its breakdown products, including ADP and adenosine, has strong implications for the regulation of immune responses and blood coagulation. Disturbances in circulating nucleotide concentrations could be indicative for different pathologies as ATP is for example released upon stroke,^8^ and ATP concentrations are high in the tumor microenvironment.^7^ There is a long history of disagreement on the “normal” levels of adenine nucleotides and efforts to control for artifacts in the determination of adenosine in plasma continue today.^19,26^ For instance, inhibitors of adenosine clearance, either alone or in combination, and specially developed syringe systems for blood collection were used.^18,19^ Yet, these studies focused mainly on the accurate quantification of adenosine. Given the opposing immunological effects of ATP and adenosine and the interconvertibility of adenine nucleotides, there is a critical need for accurate and simultaneous quantification of all these metabolites. To this end, a recently published approach implemented rapid cooling of the collected blood samples to prevent the turnover of adenine nucleotides followed by a laborious sample cleanup protocol.^17^ Applying this method, the authors highlighted the importance of temperature for ATP protection and adenine nucleotide metabolism and provided concentrations for ATP, ADP, AMP, and adenosine in plasma. However, especially in clinical setups, the rapid cooling of blood samples is challenging and might compromise the applicability of this approach. Of note, using the here described method, we obtain plasma concentrations of ATP in the range from 200 nM to 650 nM and adenosine concentrations of around 130 nM, which are very similar to previously reported data in EDTA and heparin plasma, respectively.^17^ Additionally, in line with previous observations, we also found that the concentration of adenosine was higher in heparin-containing blood collection tubes compared to EDTA-containing tubes.^17,27^ This higher concentration of adenosine in heparin tubes may result from the generation of adenosine out of ATP/ADP/AMP mediated by the activity of ENTPDases like CD39, followed by CD73, which are blocked in the EDTA tubes, as EDTA complexes with divalent cations necessary for the activity of these enzymes. Not surprisingly, the concentrations of ATP, ADP, and AMP detected in the heparin plasma were very low, indicative of their degradation. The reported inhibitory effect of heparins on ENPP1 ^28^ suggests a minor contribution of this enzyme to ATP degradation in the bloodstream. However, the inhibitory effect of heparin is still under discussion,^15^ and the use of highly specific inhibitors for ENPP1 will be crucial to clarify the exact contribution of ENPP1 to ATP catabolism.

The ATP concentrations in EDTA plasma tubes (without inhibitors) were slightly higher than those in EDTA+I tubes. This can probably be explained by the regeneration of ATP from adenosine via backward ecto-phosphotransfer reactions mediated by adenosine kinase (ADK),^24^ adenylate kinase (AK), and nucleoside diphosphate kinase (NDPK).^23^ Even though these enzymes should be blocked upon chelation of their divalent cofactors^29,30^ by EDTA, EDTA concentrations might be too low in EDTA-only tubes (3.6 mM) compared to EDTA+I tubes, where additional EDTA (15 mM) was added. Along that line, Ledderose et al. observed rising ATP levels once EDTA plasma was not immediately cooled, highlighting the importance of backward phosphotransfer reactions in the extracellular milieu.^17^

The levels of ATP metabolites determined in the serum samples were markedly different from those of all plasma samples. ATP, ADP, AMP, and adenosine were barely detectable, while the adenosine degradation products inosine, hypoxanthine, and xanthine showed the highest concentrations in the serum tube. In contrast to the preparation of plasma, blood for serum preparation is kept at room temperature, allowing coagulation. Coagulation of blood involves the activation of platelets, which is accompanied by the release of diadenosine polyphosphates (Ap_n_A).^31,32^ Ap_n_A is a pair of adenosine molecules bridged by a variable number of phosphates. After their release, Ap_n_A can be degraded by ENPP1 and CD73 to form adenosine, which may then be further metabolized. Since platelet activation only happens in serum but not in plasma samples, Ap_n_A release and degradation may be causal for the substantially higher concentrations of inosine, hypoxanthine, and xanthine in serum. Finally, as uric acid levels are mainly determined by its production in the liver, intestines, muscle, and kidney,^33^ uric acid could be detected at the same level in all blood collection tubes. Even with our small cohort, we confirmed previously reported higher levels of uric acid in males than females,^25^ underscoring the robustness of our method. Nevertheless, the sex differences reported here are limited by sample size and require evaluation in larger cohorts. Further, we detected higher concentrations of ATP, ADP, and AMP in EDTA-only plasma in men, suggesting that there might be sex-specific differences in the expression of ADK and AK,^34^ which might not be fully blocked by EDTA only. Of note, the potential overestimation of these metabolites due to insufficient blocking of ectoenzymes may even provide false positive sex differences and once more highlights the need for practical approaches preventing rapid adenine nucleotide interconversion. In line with this, one limitation of our study is that we have not specifically targeted enzymes of the ATP regeneration pathway by employing specific inhibitors such cAMP analogues to block NDPK^35^ but only used EDTA as a rather unspecific inhibitor, complexing their cofactors. In the same line, more specific and potent inhibitors for blocking CD73 ^36,37^ or ENPP1 ^38^ could be implemented. In conclusion, the inhibitor cocktail could be optimized to enhance specificity and reduce redundancy of inhibition as, e.g., EDTA already blocks many enzymes. Importantly, all inhibitors should be tested for their storage stability and suitability for LC-MS analysis.

It is very difficult to determine the “true” adenine nucleotide concentrations in blood because their generation, degradation, interconversion, and uptake constitute a highly dynamic process *in vivo* that cannot be accurately reconstructed *ex vivo*. With the workflow presented here and recommendations regarding the use of blood collection tubes, we do not claim to assess the “true” concentration of ATP or adenosine in blood, but we obtain a faithful quantification that allows us to compare physiological and disease states, which could be implemented in clinical routine.

In this study, we focused on the measurement of metabolites of the ATP to uric acid axis, but we previously established the quantification of other metabolites using the same LC-MS method, for example, of metabolites belonging to the NAD pathway.^22^ The integration of additional nucleotide derivatives of interest, for instance, cGAMP or cAMP, into the analysis workflow is readily feasible.

In conclusion, we describe a rapid and reliable approach for the simultaneous determination of ATP metabolites in human blood, which is applicable in clinical routines. Our data show that blood collection tube additives have a major effect on the concentration of ATP metabolites in human samples and that discordant results in the literature may derive from differences in sample preparation. Choosing the “appropriate” blood collection tubes is a key aspect in the planning of clinical studies. To obtain the full picture of ATP metabolites, we recommend blood collection in parallel in a serum tube and an EDTA tube containing inhibitors of ectonucleotidases and nucleoside transporters. The accurate quantification of ATP metabolites in blood, along with the comparison to established reference ranges, may help to monitor disease progression and provide insights into the health status of an individual in the future.

## Supplementary Material



## Notes

Supporting Figure 1: Sex and age distribution of the cohorts; Supporting Table 1: HPLC-MS/MS method validation parameters for xanthine and uric acid; Supporting Table 2: Concentration of ATP metabolites in plasma and serum; Supporting Figure 2: ATP metabolites in plasma and serum disaggregated by sex (PDF)
