# Supplementary material for: Reliable Determination of ATP and Its Metabolites by LC-MS Using Blood Collection Tubes with and without Ectonucleotidase Inhibitors
Source: ACS Pharmacol Transl Sci. 2025 Nov 7;8(12):4326–34. doi: 10.1021/acsptsci.5c00446 (PMC12707265; doi:10.1021/acsptsci.5c00446)
Supplement: Supplementary file 1 [file pt5c00446_si_001.pdf]

Supporting Information

Reliable determination of ATP and its metabolites by LC-MS using blood collection tubes with and without ectonucleotidase inhibitors

Riekje Winzer[1#], Johanna Hiefner[2#], Romy Hackbusch[1], Moritz A Link[3], Götz Thomalla[3], Eva Tolosa[1], Anna Worthmann[2*]

[1]Department of Immunology, University Medical Center Hamburg-Eppendorf, 20246 Hamburg, Germany.

[2]Department for Biochemistry and Molecular Biology, University Medical Center Hamburg Eppendorf, 20246 Hamburg, Germany.

[3]Department of Neurology, University Medical Center Hamburg-Eppendorf, Hamburg, 20246, Germany.

[#]Contributed equally

*Corresponding author (a.worthmann@uke.de)

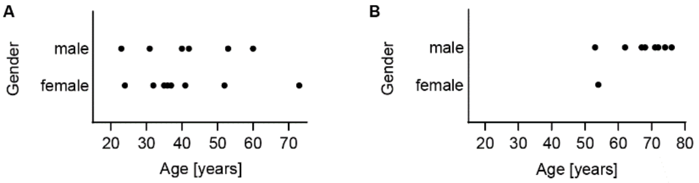

Supporting Figure 1. Sex and age distribution of the cohorts. Sex and age distribution for **A** a cohort of healthy donors (n = 15) and **B** of patients with high cardiovascular risk who were admitted to the clinic (n = 9).

Supporting Table 1. HPLC-MS/MS method validation parameters for xanthine and uric acid.

Analyte	LOD [µM]	LLOQ [µM]	Carryover [%]	Calibration range [µM]	Slope	i	R2
Xanthine	0.002	0.1	0	0.1–6	2.9022	−0.0473	0.9997
Uric acid	0.02	0.6	0	0.6–20	1.3083	−0.3214	0.9950

LOD, Limit of detection; LLOQ, Lower limit of quantification; i, Intercept; R2, Correlation coefficient.

Supporting Table 2. Concentration of ATP metabolites in plasma and serum. Metabolites of the ATP to uric acid axis were measured in different blood collection tubes by LC-MS (n = 15). Table shows the median and range (lowest and highest value) of the data shown in Figure 2B.

	EDTA	EDTA+I	Heparin	Serum
ATP	0.67 (0.19–1.60)	0.36 (0.09–0.93)	0.00 (0.00–0.08)	0.00 (0.00–0.08)
ADP	0.32 (0.00–0.99)	0.12 (0.00–0.87)	0.00 (0.00–0.28)	0.00 (0.00–0.19)
AMP	0.14 (0.00–0.76)	0.00 (0.00–0.65)	0.00 (0.00–0.04)	0.00 (0.00–0.04)
Adenosine	0.02 (0.00–0.02)	0.13 (0.09–0.28)	0.02 (0.02–0.06)	0.02 (0.02–0.05)
Inosine	0.14 (0.13–0.18)	0.21 (0.13–0.37)	0.40 (0.28–0.79)	7.09 (0.77–14.60)
Hypoxanthine	1.52 (1.04–2.71)	2.31 (1.13–6.88)	1.27 (0.57–2.61)	8.37 (3.47–17.45)
Xanthine	4.78 (2.61–8.52)	6.90 (3.53–10.34)	5.10 (2.99–8.27)	24.48 (10.18–36.74)
Uric acid	235.89 (130.83–351.61)	275.43 (139.12–476.09)	241.35 (129.15–352.77)	246.45 (144.39–472.45)

All values are given in micromolar concentration [µM], shown is median (min–max).

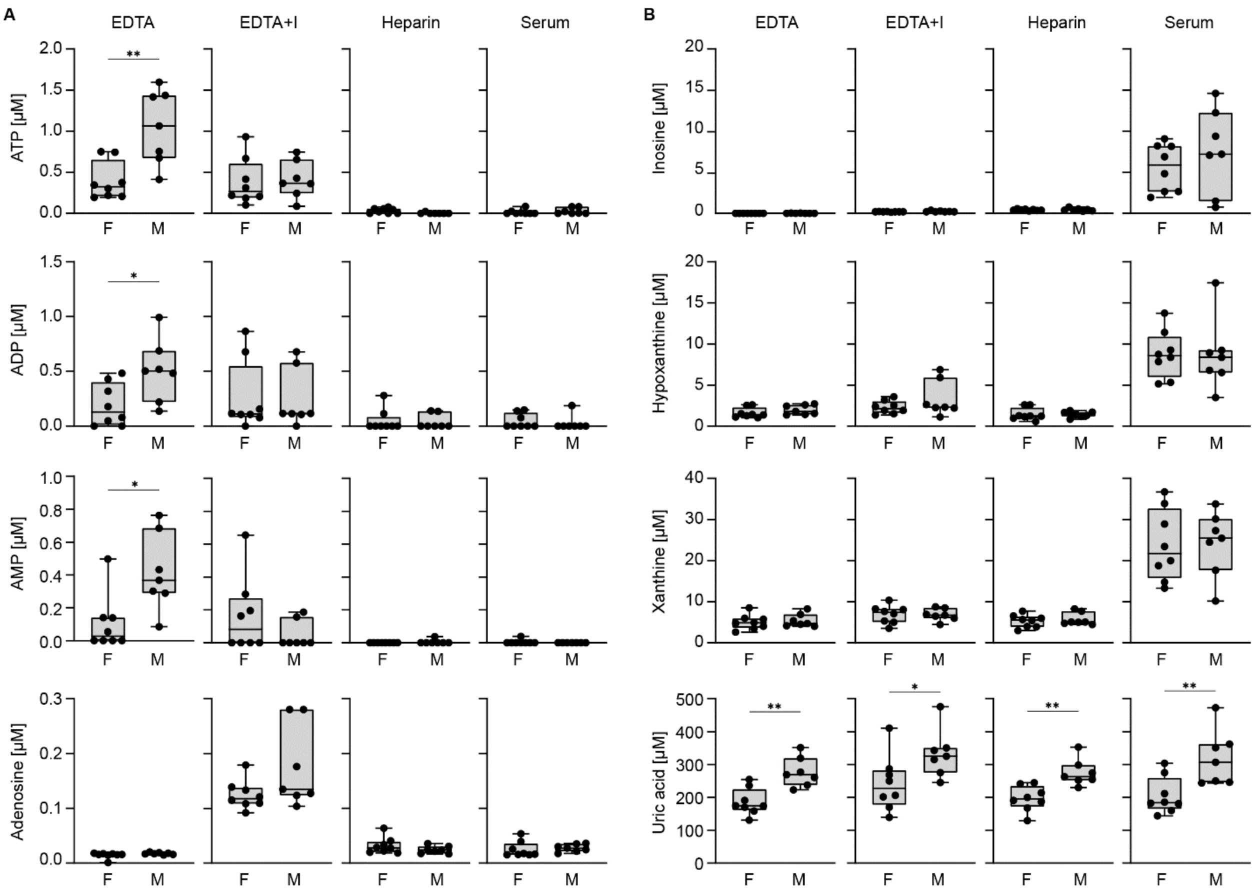

Supporting Figure 2. ATP metabolites in plasma and serum disaggregated by sex. A-B Comparison of the concentrations of metabolites of the ATP to adenosine (A) and inosine to uric acid (B) axis in female and male donors (n = 8 female and n = 7 male). Mann-Whitney test was used to compare the differences between the two groups (*$p \leq 0.05$, **$p \leq 0.01$, ***$p \leq 0.001$, ****$p \leq 0.0001$). F, Female; M, Male.
